# Role of Bempedoic Acid in Clinical Practice

**DOI:** 10.1007/s10557-021-07147-5

**Published:** 2021-04-05

**Authors:** Christie M. Ballantyne, Harold Bays, Alberico L. Catapano, Anne Goldberg, Kausik K. Ray, Joseph J. Saseen

**Affiliations:** 1 Department of Medicine, Baylor College of Medicine, One Baylor Plaza, BCM 285, Houston, TX 77030 USA; 2 Louisville Metabolic and Atherosclerosis Research Center, Louisville, KY USA; 3 Department of Pharmacological and Biomolecular Sciences, University of Milan and IRCCS Multimedica, Milan, Italy; 4 Division of Endocrinology, Metabolism and Lipid Research, Washington University School of Medicine, St. Louis, MO USA; 5 Department of Primary Care and Public Health, Imperial College London, London, UK; 6 Departments of Clinical Pharmacy and Family Medicine, University of Colorado Anschutz Medical Campus, Aurora, CO USA

**Keywords:** Atherosclerotic cardiovascular disease, ATP-citrate lyase, Bempedoic acid, High-sensitivity C-reactive protein, Hypercholesterolemia, Low-density lipoprotein cholesterol

## Abstract

Many patients do not achieve optimal low-density lipoprotein cholesterol (LDL-C) levels with statins alone; others are unable to tolerate statin therapy. Additional non-statin treatment options including ezetimibe, proprotein convertase subtilisin/kexin type 9 inhibitors, and bile acid sequestrants are often necessary to further reduce the risk of atherosclerotic cardiovascular disease. This review provides practical guidance as to the use of bempedoic acid to lower LDL-C and includes direction as to which patients may benefit and advice for safety monitoring during treatment. Bempedoic acid, a new class of agent, is a prodrug converted to bempedoyl-CoA by very long-chain acyl-CoA synthetase 1, an enzyme with high expression in the liver but that is undetectable in the skeletal muscle. Bempedoic acid inhibits the enzyme adenosine triphosphate (ATP)-citrate lyase, which lies two steps upstream from β-hydroxy β-methylglutaryl-CoA reductase in the cholesterol biosynthesis pathway. In clinical trials conducted in patients with or at risk for atherosclerotic cardiovascular disease or familial heterozygous hypercholesterolemia, bempedoic acid in combination with statins and/or ezetimibe significantly reduced LDL-C, apolipoprotein B, and high-sensitivity C-reactive protein compared with placebo. Bempedoic acid is generally well tolerated with no clinically meaningful increase in muscle-related symptoms relative to placebo, even in patients taking maximally tolerated statins. A small increase in serum uric acid (mean increase 0.8 mg/dL) is the most noteworthy adverse effect. Bempedoic acid provides an effective and generally well-tolerated medication to further reduce LDL-C in patients taking maximally tolerated statins or manage LDL-C levels in those who are unable to take statins. The potential for a reduced incidence of major cardiovascular events with bempedoic acid is being investigated in the CLEAR Outcomes trial, with results expected in 2023.

## Introduction

Lowering of low-density lipoprotein cholesterol (LDL-C) with statins reduces atherosclerotic cardiovascular disease (ASCVD) risk and associated mortality proportional to the absolute decrease in LDL-C [1]. For each 1-mmol/L (38.7-mg/dL) reduction in LDL-C, the risk of major cardiovascular events is reduced by 22% [2]. Up to 80% of patients treated with statins do not achieve an optimal LDL-C level, because of high baseline LDL-C levels, aggressive LDL-C treatment goals, lack of sufficient statin efficacy, or poor statin persistence or adherence, which is often related to adverse drug reactions [3]. Therefore, some patients may benefit from additional LDL-C-lowering therapies, such as ezetimibe or proprotein convertase subtilisin/kexin type 9 (PCSK9) inhibitors, when LDL-C is ≥70 or ≥ 100 mg/dL, despite treatment with maximally tolerated statin [4, 5].

Guidelines for cholesterol management reserve combination therapy for patients who do not achieve sufficient LDL-C lowering with statin monotherapy [4, 5]. Use of additional and effective add-on treatments in patients for whom statins alone do not sufficiently lower LDL-C levels, or alternative treatments for patients who cannot take statins, may help to reduce the risk of ASCVD. Despite this, a recent cross-sectional study of almost 6000 patients in 18 countries in the European Union found that combination of a statin with another lipid-lowering agent was low, including those patients who are at high and very high risk for ASCVD [6]. To date, add-on or alternative treatments to statins include primarily ezetimibe, PCSK9 inhibitors, and/or bile acid sequestrants, mainly because they have been proven to reduce LDL-C levels and cardiovascular events in randomized, placebo-controlled trials [7–9]. Ezetimibe and bile acid sequestrants have historically been the most commonly used alternative oral agents, with PCSK9 inhibitors requiring administration by injection. However, access to PCSK9 inhibitors is often challenging because of the requirement of prior insurance authorization, cost, and patients having LDL-C levels below the payer-specific threshold for monoclonal antibodies [10, 11].

## Bempedoic Acid

Bempedoic acid (Nexletol, Esperion Therapeutics, Inc., Ann Arbor, MI; NILEMDO, Daiichi Sankyo Europe GmbH, Pfaffenhofen, Germany) is a novel oral inhibitor of adenosine triphosphate (ATP)-citrate lyase (ACL) [12, 13]. Bempedoic acid administered as a 180-mg once-daily dose is approved in the USA and European Union as an adjuvant to maximally tolerated statin therapy to lower LDL-C for patients with ASCVD and for patients with heterozygous familial hypercholesterolemia (HeFH). In addition, bempedoic acid is approved in the European Union to treat patients who are unable to take any dose of a statin (statin intolerant) or for whom a statin is contraindicated. Bempedoic acid is also available as a fixed-dose combination drug product that consists of 180 mg of bempedoic acid and 10 mg of ezetimibe (Nexlizet, Esperion Therapeutics, Inc., Ann Arbor, MI; NUSTENDI, Daiichi Sankyo Europe GmbH, Pfaffenhofen, Germany) [14, 15]. To help clinicians understand how best to incorporate these lipid-lowering therapies into their practices, we review the mechanism of action for bempedoic acid, summarize its pharmacology and clinical data, and discuss practical information on the use of bempedoic acid in the clinical setting.

### Mechanism of Action and Pharmacodynamics

**Fig. 1:**
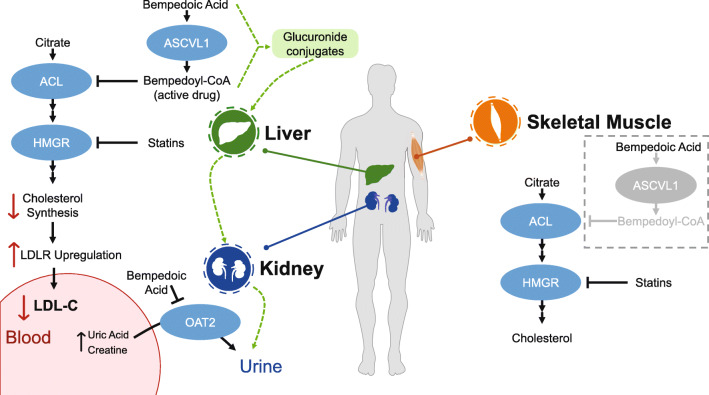
Mechanism of action and physiologic sites of bempedoic acid metabolism, activation, biologic activity, and clearance. Following oral administration, bempedoic acid is converted to its active metabolite bempedoyl-CoA by ASCVL1 in the liver. Bempedoyl-CoA inhibits the cytoplasmic ACL enzyme, which converts citrate to acetyl-CoA in the cholesterol synthesis pathway, leading to upregulation of LDLR. ASCVL1 is not present in the muscle so is not converted to bempedoyl-CoA. Clearance of bempedoic acid and bempedoyl-CoA is primarily enacted by glucuronidation and subsequent renal elimination. Bempedoic acid is a weak inhibitor of OAT2, resulting in minor increases in plasma uric acid and creatinine. *ACL* ATP-citrate lyase, *ASCVL1* very long-chain acyl-CoA syntheasae-1, *HMGR* 3-hydroxy-3-methylglutarate-CoA reductase, *LDL-C* low-density lipoprotein cholesterol, *LDLR* low-density lipoprotein receptor, *OAT2* organic anion transporter-2

Key physiologic sites of bempedoic acid metabolism, activation, biologic activity, and clearance are shown in Fig. 1. Bempedoic acid is a prodrug that requires conversion to the active form, bempedoyl CoA, by the enzyme very long-chain acyl-CoA synthease-1 (ACSVL1) [16, 17]. The ACSVL1 enzyme is expressed mainly in the liver and kidney, but not in the skeletal muscle or other tissues; therefore, the activity of bempedoic acid is restricted almost exclusively to the liver [16, 17]. By contrast, the activity of statins in muscle potentially contributes to statin-associated muscle symptoms that occur in some patients, resulting in poor treatment adherence and/or restricting use to low- or moderate-intensity doses [18].

Acetyl-CoA is critical for energy production; protein and histone acetylation; and synthesis of fatty acids, steroid hormones, and cholesterol. ACL is a cytosolic enzyme that cleaves mitochondria-derived citrate into oxaloacetate and acetyl-CoA. The lipid-lowering effects of bempedoyl-CoA are mediated through the inhibition of ACL in the liver, decreasing the activity of the lipid biosynthetic pathway of acetyl-CoA [16]. ACL acts two steps upstream of 3-hydroxy-3-methylglutaryl (HMG)-CoA reductase in this pathway [19]. By inhibiting cholesterol synthesis in the liver, bempedoyl-CoA induces the upregulation of the LDL receptor and stimulates the uptake of LDL particles by the liver, which in turn contributes to the reduction of LDL in the blood [20–22].

Further, bempedoyl-CoA is not found in the plasma of individuals treated with bempedoic acid and likely does not escape the hepatocyte, suggesting that its activity is limited to the liver. Because bempedoyl-CoA has a unique mechanism of action compared with other lipid-lowering therapies, it offers the opportunity for complementary LDL-C lowering with concomitant statin, ezetimibe (blocking intestinal cholesterol absorption), and/or PCSK9 inhibitor (preventing LDL receptor degradation) therapy.

In preclinical animal models, free bempedoic acid increased the activity of AMP-activated protein kinase (AMPK) [16]. This enzyme catalyzes regulatory phosphorylation of a number of substrates involved in inflammatory signaling and lipid metabolism [23, 24]. Results from studies using an apolipoprotein E (ApoE)/AMPK β1 double knockout mouse model suggest this activity is not responsible for the LDL-C-lowering effects of bempedoic acid [17]. AMPK is also implicated in carbohydrate metabolism and may have a role in the progression to diabetes and obesity [23, 24]. However, the clinical relevance of possible bempedoic acid stimulation of AMPK in humans is not clear.

### Pharmacology

Following multiple-dose administration, mean (standard deviation [SD]) maximum plasma concentration (*C*_max_) was 20.6 (6.1) μg/mL and the area under the curve (AUC) was 289.0 (96.4) μg•h/mL. The median time to reach *C*_max_ of bempedoic acid is 3.5 h, with steady state reached after 7 days. Food has no effect on the oral bioavailability of bempedoic acid, and its pharmacokinetic (PK) properties are not affected by age, sex, race, or weight.

Bempedoic acid is approximately 99% protein bound in plasma, is not found in blood cells, and has a volume of distribution of 18 L consistent with a minimal extrahepatic distribution. The major metabolites of bempedoic acid, found in the plasma, are the glucuronides of bempedoic acid and bempedoyl-CoA. In vitro, glucuronidation occurs via the enzyme UDP-glucuronosyltransferase-2B7 (UGT2B7) found in the liver, kidneys, and lower gastrointestinal tract. The elimination of bempedoic acid occurs primarily in the kidneys, with 70% recovered in urine and 30% in feces. After once-daily dosing, the steady-state clearance of bempedoic acid is 11.2 mL/min. The major metabolite found in urine is the acyl glucuronide conjugate, with less than 2% being recovered as unmetabolized bempedoic acid.

In a single-dose pharmacokinetic study in patients with varying degrees of renal function (based on estimated glomerular filtration rate [eGFR]), the AUC for bempedoic acid was increased 1.5-, 2.3-, and 2.4-fold in patients with mild (eGFR ≥60–89 mL/min), moderate (eGFR >30–59 mL/min), or severe (eGFR ≤30 mL/min) renal impairment, respectively, compared with patients with normal renal function. However, the number of participants in each group was quite small (with five to eight participants in each group). In a population PK analysis of data from clinical trials (*n* = 2261), exposure was increased 1.4- and 1.9-fold in patients with mild or moderate renal impairment, respectively. For patients with mild or moderate hepatic impairment, bempedoic acid exposure was decreased by <22%. AUC for the active bempedoyl-CoA metabolite was decreased by 23% and 36% in patients with mild or moderate hepatic impairment, respectively, compared with patients with normal hepatic function. These PK changes in patients with renal and hepatic impairment are not clinically significant and are not expected to affect the efficacy or safety profile of bempedoic acid; no dosage adjustment is necessary. Bempedoic acid has not been studied in patients with more severe kidney and liver disease, including patients with end-stage renal disease and Child-Pugh Class C hepatic impairment.

### Drug-Drug Interactions

Neither bempedoic acid, its active metabolite bempedoyl-CoA, nor the glucuronide forms are metabolized or interact with cytochrome P450 enzymes including CYP3A4 and CYP2C9. As a result, drug-drug interactions with drugs metabolized by this route, including warfarin, are not anticipated.

Results from in vitro studies have demonstrated that bempedoic acid glucuronide is a substrate for the organic anion transporter (OAT) 3, which transports smaller hydrophilic organic anions [25]; bempedoic acid weakly inhibits this transporter at concentrations well above the clinically relevant range. Co-administration of bempedoic acid with the OAT blocker probenecid results in moderate increases in the AUC and *C*_max_ of bempedoic acid and bempedoyl-CoA following a single dose, but these changes were not considered clinically meaningful based on the current understanding of bempedoic acid exposure response for efficacy and safety [26]. Bempedoic acid and its glucuronide also weakly inhibit the organic anion transporting polypeptides (OATPs) 1B1 and OATP1B3, which typically transport large hydrophobic organic anions [25], at clinically relevant concentrations, and also weakly inhibit OAT2 in vitro. The weak inhibition of OAT2 is the likely mechanism responsible for minor elevations in serum creatinine and uric acid levels observed in patients treated with bempedoic acid [27]. Although bempedoic acid is a weak inhibitor of OAT2, significant drug-drug interactions are not anticipated.

The most important drug-drug interactions involving bempedoic acid are those between bempedoic acid and simvastatin and pravastatin. Co-administration of simvastatin 20 mg with bempedoic acid 240 mg, or simvastatin 40 mg with bempedoic acid 180 mg, in healthy participants resulted in an approximately 2-fold and 1.5-fold increase in simvastatin AUC and *C*_max_, respectively. A similar 2-fold increase in AUC and *C*_max_ was seen when pravastatin 40 mg was dosed with steady-state bempedoic acid 240 mg in healthy participants. These interactions are important, given that many patients taking bempedoic acid also take statins concomitantly, including patients who are intolerant of higher-intensity statin therapy; therefore, doses of simvastatin >20 mg and pravastatin >40 mg should be avoided. The interaction of bempedoic acid with pravastatin may be due to the effects of bempedoic acid on the OATP2 transporter, which transports pravastatin [28]. However, the mechanism underlying the interaction of bempedoic acid with simvastatin (which is metabolized by CYP3A4) is less clear, but is not believed to be via cytochrome P450 enzymes as bempedoic acid and its active metabolite do not appear to interact with cytochrome P450 enzymes. A 1.7-fold increase in AUC of the statins atorvastatin and rosuvastatin and/or their major metabolites when taken with bempedoic acid has been observed. In addition, co-administration of bempedoic acid with high-dose atorvastatin 80 mg in patients with hypercholesterolemia resulted in changes <30% in AUC and *C*_max_ of atorvastatin and its main metabolite ortho-hydroxy atorvastatin [29]. Taken together, this suggests only a weak interaction between bempedoic acid and atorvastatin or rosuvastatin; no dose adjustments are required when bempedoic acid is co-administered with atorvastatin or rosuvastatin.

Increases in AUC and *C*_max_ of ezetimibe were < 20% following a single dose when added to steady-state bempedoic acid. After accounting for the pharmacologically active glucuronide form of ezetimibe, overall AUC and *C*_max_ were increased by approximately 1.6- and 1.8-fold, respectively. These changes in the PK of ezetimibe when given concomitantly with bempedoic acid are not clinically meaningful and do not affect dosing recommendations, as demonstrated by the clinical activity and safety of the bempedoic acid and ezetimibe fixed-dose combination compared with either agent alone [30]. Further drug interaction studies have shown that bempedoic acid has no effect on the PK of either metformin [31] or an oral contraceptive containing norethindrone 1 mg plus ethinyl estradiol 0.035 mg [32].

## Bempedoic Acid Phase 3 Clinical Trials Summary

**Fig. 2:**
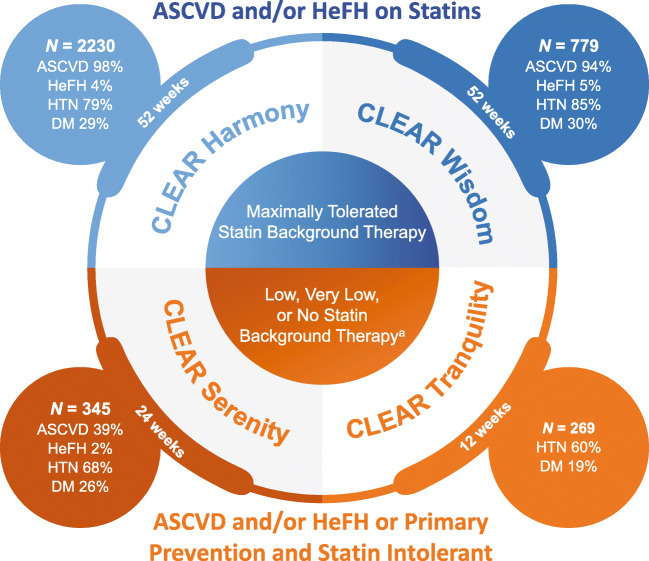
Overview of bempedoic acid phase 3 program [33–36]. ^a^Low-dose statin therapy = average daily dose of rosuvastatin 5 mg, atorvastatin 10 mg, simvastatin 10 mg, lovastatin 20 mg, pravastatin 40 mg, fluvastatin 40 mg, or pitavastatin 2 mg. Average daily doses less than these were considered very low-dose statin therapy. *ASCVD* atherosclerotic cardiovascular disease, *DM* diabetes mellitus, *HeFH* heterozygous familial hypercholesterolemia, *HTN* hypertension

In the CLEAR Harmony (NCT02666664) and CLEAR Wisdom (NCT02991118) trials, patients with ASCVD or HeFH who were at high cardiovascular risk and who were receiving maximally tolerated statin therapy were randomized to receive bempedoic acid or placebo (ASCVD and/or HeFH on statins pool) (Fig. 2) [33, 34]. ASCVD was defined as documented coronary heart disease (acute myocardial infarction [MI], silent MI, unstable angina, coronary revascularization, or clinically significant coronary heart disease diagnosed by invasive or non-invasive testing), or coronary heart disease risk equivalents (cerebrovascular atherosclerotic disease and peripheral arterial disease). In the CLEAR Harmony and CLEAR Wisdom trials, 28.6% and 30.3% of patients also had diabetes, and 80% of patients in both trials had hypertension; approximately 50% of patients were receiving a high-intensity statin.

In two additional phase 3 studies (CLEAR Serenity [NCT02988115] and CLEAR Tranquility [NCT03001076]), patients who had a history of not being able to take statins at more than the lowest approved daily dose were randomized to receive bempedoic acid or placebo (statin intolerant pool) [35, 36]. Patients included in these trials were being treated for primary and secondary prevention of cardiovascular disease (CVD). In the CLEAR Tranquility trial, patients in both treatment arms were on background ezetimibe and were unable to take more than low-dose statin therapy. Patients in the CLEAR Serenity trial had been unable to take at least two statins, one at low dose, because of a prior adverse event (AE) that started or increased during statin therapy and resolved or improved when statin therapy was discontinued. As a result, no patients in these 2 trials were receiving moderate- or high-intensity statin treatment.

### LDL-C Lowering

**Fig. 3:**
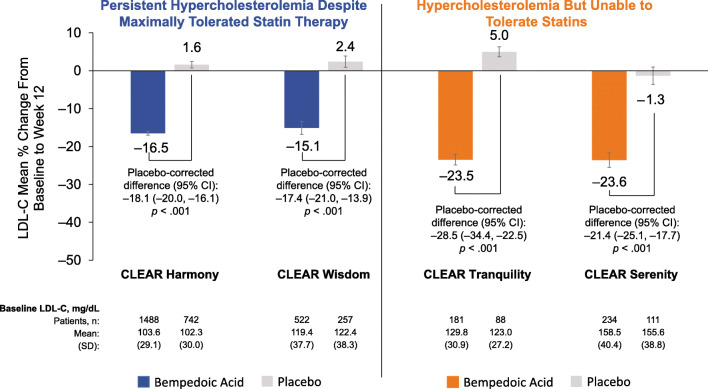
Effect of bempedoic acid on LDL-C after 12 weeks of treatment [33–36]. *CI* confidence interval, *LDL-C* low-density lipoprotein cholesterol

Baseline LDL-C levels across these four trials ranged from 103.2 to 157.6 mg/dL [33–36]. Despite the different patient populations enrolled, bempedoic acid was consistently associated with significant decreases in LDL-C at week 12, compared with essentially no change in the placebo arm (*p* < 0.001 in all trials; Fig. 3). In the two trials of bempedoic acid added to stable maximally tolerated lipid-lowering therapy (ASCVD and/or HeFH on statins pool), the placebo-corrected change in LDL-C from baseline at week 12 was a decrease of 17.4% to 18.1% [33, 34]. Patients with HeFH made up only a minority of patients in these trials. In an early analysis, patients with HeFH had a greater placebo-corrected change in LDL-C of −22.3% (*n* = 112) compared with −18.3% among those patients without a diagnosis of HeFH (*n* = 2897) [37].

In the two trials conducted in patients taking low dose or no statin (statin intolerant pool), the placebo-corrected change in LDL-C from baseline was a decrease at week 12 of 21.4% to 28.5% [35, 36]. These improvements in LDL-C were sustained through 24 to 52 weeks of follow-up [33–35]. Similar patterns were observed for changes in other lipid parameters, including non-high-density lipoprotein cholesterol, total cholesterol, and apolipoprotein B (Table 1). Taken together, these data demonstrate that bempedoic acid is an effective treatment option to lower atherosclerotic LDL-C either in combination with maximally tolerated lipid-lowering therapy in patients with ASCVD and/or HeFH or as monotherapy in patients who are unable to take statin therapy.

**Table 1:** Effect of bempedoic acid on other lipid parameters [39]

Endpoint	Placebo-corrected difference in LS mean percent change from baseline at week 12 (95% CI)	
	ASCVD/HeFH on statins pool	Statin intolerant pool
Non-HDL-C	−13.1% (−14.7 to −11.6) *p* < 0.001	−20.4% (−23.4 to −17.5) *p* < 0.001
TC	−11.1% (−12.2 to −9.9) *p* < 0.001	−16.2% (−18.4 to −13.9) *p* < 0.001
ApoB	−12.1% (−13.6 to −10.7) *p* < 0.001	−16.9% (−19.6 to –14.2) *p* < 0.001

*ApoB* apolipoprotein B, *95% CI* 95% confidence interval, *non-HDL-C* non-high-density lipoprotein cholesterol, *LS* least squares, *TC* total cholesterol

### Reductions in High-Sensitivity C-Reactive Protein

**Fig. 4:**
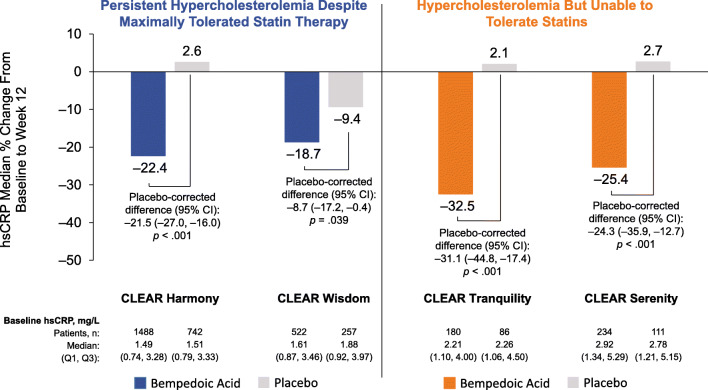
Effect of bempedoic acid on hsCRP after 12 weeks of treatment [33–36]. *CI* confidence interval, *hsCRP* high-sensitivity C-reactive protein

Elevated high-sensitivity C-reactive protein (hsCRP) is an established prognostic marker of future coronary events. As shown in Fig. 4, addition of bempedoic acid was associated with a consistent statistically significant decrease in hsCRP compared with placebo across all four pivotal phase 3 trials [33–36]. This reduction suggests the possibility that bempedoic acid has anti-inflammatory effects; however, evaluation of the clinical relevance of any anti-inflammatory effects awaits further confirmation in future studies.

### Combination with Ezetimibe

Ezetimibe is used as an add-on therapy to statins, or as an alternative for patients who are unable to take statins [38]. To that end, another phase 3 trial, which included patients at high CVD risk due to the presence of ASCVD, multiple CVD risk factors, or HeFH, investigated the efficacy and safety of a bempedoic acid and ezetimibe fixed-dose combination [30]. A potential benefit of such a combination is that it may help to improve outcomes by facilitating better treatment persistence [38]. The study included four treatment arms: bempedoic acid and ezetimibe fixed-dose combination, bempedoic acid alone, ezetimibe alone, and placebo. Overall, 46.5% of patients in this trial had diabetes, and 85.4% had hypertension. The bempedoic acid and ezetimibe fixed-dose combination decreased LDL-C by 36.2% at week 12, as compared with a 23.2% decrease with ezetimibe alone, 17.2% with bempedoic acid alone, and a 1.8% increase with placebo. Ezetimibe used alone in this study had only a small effect on hsCRP (8.2% decrease) at 12 weeks, compared with a 31.9% decrease in hsCRP with bempedoic acid alone. A 35.1% decrease in hsCRP was observed at 12 weeks with the bempedoic acid and ezetimibe fixed-dose combination; patients in the placebo arm had a 21.6% increase in hsCRP. The fixed-dose combination of bempedoic acid and ezetimibe provides for substantial reductions in LDL-C in patients taking maximally tolerated statin therapy.

### Common Adverse Events

The most common treatment-emergent AEs, regardless of causality, reported in the four phase 3 bempedoic acid clinical trials were nasopharyngitis, urinary tract infection, and arthralgia, all occurring at a lower frequency in patients taking bempedoic acid than in patients receiving placebo [39]. This safety profile was consistent when bempedoic acid was given alone or in combination with maximally tolerated statin or other lipid-lowering therapy (e.g., ezetimibe). The study assessing the bempedoic acid and ezetimibe fixed-dose combination did not reveal any new safety concerns, with an AE profile similar to that of either of the two agents alone or to placebo, and consistent with results from safety reports of other trials with bempedoic acid [30]. AEs of special interest are discussed in further detail below.

## Special Topics and Clinical Considerations

Results from the four phase 3 clinical trials have demonstrated that bempedoic acid is effective in lowering LDL-C and is generally well tolerated. Bempedoic acid can be considered for several clinical scenarios including:

- Patients with HeFH and/or ASCVD who are taking maximally tolerated statin therapy with or without ezetimibe but whose LDL-C remains high (US and EU indication)
- Patients with HeFH and/or ASCVD, but who have a history of not being able to take a high-dose statin and are not willing or able to receive treatment with a PCSK9 inhibitor (EU indication)
- Patients with high LDL-C or ASCVD but who are not receiving a statin who may be candidates for a combination of bempedoic acid and ezetimibe (EU indication)

**Fig. 5:**
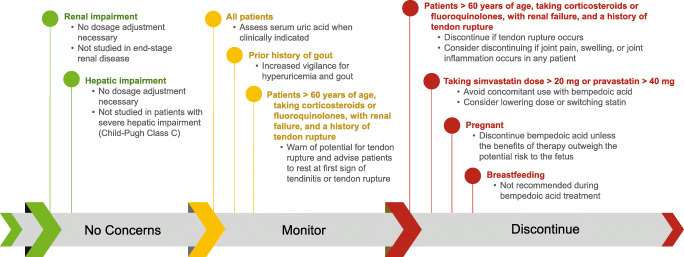
Management of patients receiving bempedoic acid therapy

Below, we discuss important topics to be addressed when providers consider the addition of bempedoic acid to their patients’ regimens to treat hypercholesterolemia, including scenarios in which bempedoic acid should be used with heightened awareness **(**Fig. 5**)**.

### Use in Combination with Statins

Overall, in the phase 3 studies, bempedoic acid was not associated with a clinically meaningful increase in the incidence of muscle-related AEs commonly reported with statin use, including myalgia and muscle weakness [40]. Among patients in the ASCVD and/or HeFH on statins pool, 51% of whom were taking high-intensity statins; muscle spasms (bempedoic acid, 3.6% and placebo, 2.3%), pain in the extremity (bempedoic acid, 3.0% and placebo, 1.7%), myalgia (bempedoic acid, 5.2% and placebo, 5.3%), and muscle weakness (bempedoic acid, 0.5% and placebo, 0.5%) were reported at the same incidence rate in patients treated with bempedoic acid or placebo [33, 34]. For patients with a history of not being able to take a statin, muscle-related symptoms (including myalgia and muscle weakness) were not increased with bempedoic acid relative to placebo [35, 36].

In the phase 3 development program involving over 3000 patients, no instances of myopathy or rhabdomyolysis were reported. Over 90% of the patients who could not take statins and were enrolled in CLEAR Serenity had experienced muscle symptoms during prior statin treatment [35]. The lack of additional muscle symptoms with bempedoic acid when added to statins, even when bempedoic acid further lowered LDL-C, may be due to its distinct mechanism of action as compared with statins, and possibly because of a lack of exposure to the active bempedoyl-CoA metabolite in muscle tissue [17].

#### Clinical Considerations

Despite the demonstrated efficacy of bempedoic acid in combination with high-dose statins, co-administration of bempedoic acid with doses of simvastatin >20 mg or pravastatin >40 mg should be avoided as it results in an approximate 2-fold increase in statin levels. For patients receiving simvastatin or pravastatin above these doses, consider lowering the dose of the statin or switching to a comparable dose (or lower dose) of an alternative statin, such as atorvastatin or rosuvastatin.

### Elevated Uric Acid Levels

Bempedoic acid increases the incidence of elevated uric acid, which in some patients can lead to hyperuricemia or gout. The mechanism of this increase of uric acid is believed to be due to competition between uric acid and the bempedoic acid glucuronide metabolite for the same renal OAT2 transporter [33, 41].

In the phase 3 studies, increases in blood uric acid levels occurred in 2.1% of patients treated with bempedoic acid compared with 0.5% of those receiving placebo, with hyperuricemia reported in 1.7% and 0.6% of patients treated with bempedoic acid or placebo, respectively [39]. Increases in uric acid were reversible on discontinuation of study drug. Development of acute gout occurred in 1.4% and 0.4% of patients, respectively. The mean increase in uric acid at 12 weeks was 0.82 mg/dL for bempedoic acid vs −0.02 mg/dL for placebo. This increase was apparent within 4 weeks of treatment, was stable over time, and was reversible after withdrawal of drug [40]. A history of gout also appeared to increase the risk of gout with bempedoic acid; 14 (11.0%) of 127 patients with a history of gout developed acute gout while they received bempedoic acid compared with 2 (2.9%) of 69 patients with a history of gout who received placebo. In contrast, 0.8% of patients with no history of gout developed the condition while taking bempedoic acid (compared with 0.3% with placebo).

#### Clinical Considerations

Patients who are at risk for hyperuricemia or acute gout (i.e., those with elevated uric acid levels or history of gout) warrant heightened vigilance and additional monitoring while they are taking bempedoic acid. Analyses of shorter-term clinical trials suggest that patients who, at baseline, have no history of gout and no elevations in uric acid have an incidence of subsequent gout similar to that of placebo [40]. According to the prescribing information, uric acid levels should be assessed periodically as clinically indicated, patients should be monitored for signs and symptoms of hyperuricemia, and urate-lowering drugs should be initiated as appropriate.

### Potential for Tendon Rupture

Findings from the phase 3 clinical trials identified some rare AEs that occurred more frequently with bempedoic acid than with placebo, although the mechanism of these events and their clinical significance are unclear. Based on a broad set of diagnostic criteria set by the US Food and Drug Administration and used for the US product label, “tendon rupture or injury” (including tendon rupture, rotator cuff syndrome, biceps tendon injury, and Achilles tendon injury) occurred in 10 (0.5%) patients treated with bempedoic acid (*n* = 2009) and no patients receiving placebo (*n* = 999) among those in the ASCVD and/or HeFH on statins pool [40]. All of the tendon ruptures or injuries occurred in patients taking moderate- or high-dose statins. No tendon ruptures or injuries were reported among patients in the statin intolerant pool who were treated with bempedoic acid and received low, very low, or no statins (*n* = 415). For the overall safety population (*n* = 3621), the frequency of tendon rupture (as defined by the Medical Dictionary of Regulatory Activities [MedDRA] preferred term) was lower, occurring in 6 (0.2%) of 2424 patients who received bempedoic acid and none of the 1197 patients who received placebo (nominal *p* = 0.19) [39].

#### Clinical Considerations

Although tendon rupture is a potentially serious AE, hypercholesterolemia (particularly in patients with HeFH) alone is associated with an increased risk of tendon rupture [42]. Other risk factors for tendon rupture include statin use [42, 43], fluoroquinolone or systemic corticosteroid use, diabetes, gout, rheumatoid arthritis, renal failure, patients who are aged older than 60 years, being male, and history of tendon disorders [43–47]. In the phase 3 bempedoic acid trials, all the patients who developed tendon rupture or injury had one or more of these risk factors, including concomitant statins [40].

Given the apparent risks, patients should be told to rest at the first sign of tendinitis and/or tendon rupture; symptoms may include any pain or swelling in the arm, shoulder, back, or ankle. Bempedoic acid should be discontinued if tendon rupture occurs. Patients already at increased risk for tendon rupture should be monitored closely as rupture may occur more frequently in these patients. Patients should be instructed to contact their healthcare provider if symptoms of gout or tendon rupture occur.

### Other Rare Adverse Events

The other rare AEs, regardless of causality, that were reported in the phase 3 studies more frequently with bempedoic acid than placebo included benign prostatic hyperplasia (BPH) or prostate enlargement and atrial fibrillation. Among patients in CLEAR Harmony and CLEAR Wisdom (ASCVD and/or HeFH on statins pool) trials, new-onset BPH or prostate enlargement was reported by 1.3% of men treated with bempedoic acid and 0.1% of men receiving placebo at a mean of 6 months after starting treatment. No cases of new-onset BPH were reported among patients in CLEAR Tranquility and CLEAR Serenity (statin intolerant pool) trials, possibly because these studies were of shorter duration. Among patients in the ASCVD and/or HeFH on statins pool, atrial fibrillation was reported in 1.7% of patients randomized to bempedoic acid and 1.1% of patients receiving placebo.

#### Clinical Considerations

The reports of BPH, prostate enlargement, and atrial fibrillation were rare in the phase 3 studies. These conditions are common among older patients (the mean age in these trials ranged from 63.8 to 65.8 years); routine monitoring for BPH or prostate enlargement and atrial fibrillation should be part of standard practice for patients with hypercholesterolemia who are receiving lipid-lowering therapy, especially among older patients.

### Laboratory Assessments

Decreases in hemoglobin levels were reported in some patients taking bempedoic acid. In the ASCVD and/or HeFH on statins pool of patients who were taking maximally tolerated statin (CLEAR Harmony and CLEAR Wisdom trials), 103 patients (4.9%) had decreased hemoglobin levels ≥2 g/dL and less than the lower limit of normal while taking bempedoic acid compared with 23 patients (2.2%) taking placebo [40]. In the statin intolerant pool (CLEAR Tranquility and CLEAR Serenity trials), similar decreases in hemoglobin levels occurred in nine (5.0%) patients treated with bempedoic acid compared with none taking placebo. The decrease in hemoglobin levels in patients receiving bempedoic acid was apparent within the first 4 weeks of treatment, was stable over time, and was reversible on discontinuation of bempedoic acid.

In addition to increases in uric acid levels, bempedoic acid has been associated with small shifts in other laboratory tests relating to potential effects on the liver, kidney, and muscle (Table 2). No patients who received bempedoic acid had a total bilirubin level > 2 times the upper limit of normal [40]. Small increases in mean blood urea nitrogen (< 2 mg/dL at week 12) and creatinine (< 0.05 mg/dL at week 12) levels in patients treated with bempedoic acid were reported within the first 4 weeks of treatment but were stable and reversible when patients discontinued treatment. The mechanism for the small increase in blood urea nitrogen levels is unknown, but as with the increase in uric acid levels, the increase in creatinine levels is thought to be due to inhibition of OAT2. The changes in liver aminotransferase, creatinine, blood urea nitrogen, and other laboratory values are consistent with results reported in previous studies of patients receiving statins [48, 49].

**Table 2:** Effect of bempedoic acid on select laboratory values of interest (overall pool) [40]

Parameter	Exposure-adjusted incidence per 100 person-years (*n*)			
	ASCVD/HeFH on statins pool		Statin intolerant pool	
	Bempedoic acid (*n* = 2009)	Placebo (*n* = 999)	Bempedoic acid (*n* = 415)	Placebo (*n* = 198)
ALT and/or AST > 3 × ULN^a^	0.6 (13)	0.3 (3)	2.8 (5)	0
ALT and/or AST > 5 × ULN^a^	0.2 (4)	0.2 (2)	1.1 (2)	0
Blood creatinine levels increased	0.9 (16)	0.4 (4)	1.8 (3)	0
Blood urea levels increased	0.2 (3)	0.1 (1)	0	0
Blood uric acid levels increased	1.8 (33)	0.4 (4)	10.7 (18)	2.4 (2)
Creatine kinase >5 × ULN^a^	0.3 (7)	0.2 (2)	0.6 (1)	0

*ALT* alanine aminotransferase, *AST* aspartate aminotransferase, *ULN* upper limit of normal

^a^Patients with repeated and confirmed elevations in aminotransferase or creatine kinase levels

#### Clinical Considerations

Mild reductions in hemoglobin levels have been associated with bempedoic acid treatment, with a small number of patients developing associated clinical symptoms [40]. The mechanism of reduced hemoglobin levels in these patients is unknown. Patients with lower hemoglobin levels may require increased scrutiny for 4 to 12 weeks after initiation of bempedoic acid and then annually to monitor for any decreases in hemoglobin levels.

The prescribing information does not require routine monitoring of hemoglobin, total bilirubin, blood urea nitrogen, creatinine, or liver enzyme levels, and, thus, these laboratory abnormalities should be monitored as clinically indicated.

### Use in Patients at High Risk for Type 2 Diabetes Mellitus

Some patients with hypercholesterolemia may also have risk factors for type 2 diabetes, including body mass index ≥30 kg/m^2^, fasting blood glucose levels ≥100 mg/dL, metabolic syndrome, or HbA1c ≥ 6% [4]. The incidence of new-onset or worsening diabetes was lower in the bempedoic acid group than in the placebo group; overall, cases of new or worsening diabetes occurred in 4.0% of bempedoic acid–treated patients compared with 5.6% of placebo-treated patients (overall safety pool, nominal *p* < 0.05) [39]. By contrast, statins have been associated with an increased incidence of new-onset diabetes compared with placebo [50–53].

### Cost-Effectiveness

The cost-effectiveness of bempedoic acid in different countries will depend on the different healthcare systems, drug pricing, and populations within each country. Results of the CLEAR Outcomes clinical endpoint study [54] will be required to establish the true cost-effectiveness of bempedoic acid. The only cost-effectiveness analysis for bempedoic acid conducted to date was based on an Australian healthcare model [55]; however, the Institute for Clinical Economic Review (ICER) has announced that a cost-effectiveness analysis for bempedoic acid in the USA is ongoing and should be available in early 2021.

## Conclusions

Bempedoic acid reduces LDL-C when combined with a statin. Bempedoic acid is also effective alone or in combination with ezetimibe, which includes a bempedoic acid plus ezetimibe fixed-dose combination tablet. In addition to its effects on LDL-C, bempedoic acid also appears to reduce hsCRP, a marker of systemic inflammation associated with ASCVD. Although the results of clinical trials focused on surrogate endpoints have been encouraging, results from the CLEAR Outcomes trial (NCT02993406) are required to confirm the clinical benefits of bempedoic acid. This ongoing trial randomized more than 14,000 patients with a history of possible statin-associated side effects, and who have established CVD or are at high risk for CVD, to receive either bempedoic acid or placebo. The CLEAR Outcomes trial will evaluate the incidence of major cardiovascular events in both groups [54], and results are expected to be reported in 2023.

In this review, we aimed to highlight key management criteria to ensure safe and effective use of bempedoic acid. Bempedoic acid is generally well tolerated with few causes for concern in most patients. The risk for AEs can be minimized through routine blood monitoring and heightened vigilance in those patients identified as being at increased risk. Bempedoic acid is not metabolized to its active form in muscle, potentially limiting muscle-related side effects. As a result, bempedoic acid may offer an additional treatment option for patients with persistent elevations in LDL-C despite the use of maximally tolerated statins and ezetimibe. Bempedoic acid provides an important addition to the armamentarium for the management of patients with less-than-optimal control of LDL-C.
