# Correction to: Role of Bempedoic Acid in Clinical Practice

**DOI:** 10.1007/s10557-021-07188-w

**Published:** 2021-04-19

**Authors:** Christie M. Ballantyne, Harold Bays, Alberico L. Catapano, Anne Goldberg, Kausik K. Ray, Joseph J. Saseen

**Affiliations:** 1 Department of Medicine, Baylor College of Medicine, One Baylor Plaza, BCM 285, Houston, TX 77030 USA; 2 Louisville Metabolic and Atherosclerosis Research Center, Louisville, KY USA; 3 Department of Pharmacological and Biomolecular Sciences, University of Milan and IRCCS Multimedica, Milan, Italy; 4 Division of Endocrinology, Metabolism and Lipid Research, Washington University School of Medicine, St. Louis, MO USA; 5 Department of Primary Care and Public Health, Imperial College London, London, UK; 6 Departments of Clinical Pharmacy and Family Medicine, University of Colorado Anschutz Medical Campus, Aurora, CO USA

**Correction to: Cardiovascular Drugs and Therapy (2021)**

10.1007/s10557-021-07147-5

The article “Role of Bempedoic Acid in Clinical Practice” written by Christie M. Ballantyne, Harold Bays, Alberico L. Catapano, Anne Goldberg, Kausik K. Ray, and Joseph J. Saseen was originally published Online First without Open Access. After publication, the author decided to opt for Open Choice and to make the article an Open Access publication. Therefore, the copyright of the article has been changed to ©The Author(s) 2021 and the article is forthwith distributed under the terms of the Creative Commons Attribution 4.0 International License (http://creativecommons.org/licenses/by/4.0/), which permits use, duplication, adaptation, distribution, and reproduction in any medium or format, as long as you give appropriate credit to the original author(s) and the source, provide a link to the Creative Commons license, and indicate if changes were made.

The original article has been corrected.

Open Access This article is distributed under the terms of the Creative Commons Attribution 4.0 International License (http://creativecommons.org/licenses/by/4.0/), which permits unrestricted use, distribution, and reproduction in any medium, provided you give appropriate credit to the original author(s) and the source, provide a link to the Creative Commons license, and indicate if changes were made. The images or other third party material in this article is included in the article’s Creative Commons licence, unless indicated otherwise in a credit line to the material. If material is not included in the article’s Creative Commons licence and your intended use is not permitted by statutory regulation or exceeds the permitted use, you will need to obtain permission directly from the copyright holder. To view a copy of this licence, visit http://creativecommons.org/licenses/by/4.0/.

